# Social Media Challenge Gone Wrong: A Severe Case of Colonic Obstruction Secondary to Xylophagia and Literature Review

**DOI:** 10.7759/cureus.92527

**Published:** 2025-09-17

**Authors:** Diego Prentice-Webb, Nikita Rao, Alejandro E Rodulfo

**Affiliations:** 1 Psychiatry, Memorial Healthcare System, Hollywood, USA; 2 Psychiatry, Schmidt College of Medicine, Florida Atlantic University, Boca Raton, USA

**Keywords:** adolescent mental health, effects of social media, hemicolectomy, intestinal obstruction, pica disorder, xylophagia

## Abstract

Pica is characterized by the persistent craving and consumption of non-food substances. In some cases, individuals ingest paper-based materials, a behavior known as xylophagia, which may lead to gastrointestinal complications if the behavior becomes severe. Social media has recently been recognized as a potential influence on emerging patterns of unusual behaviors in adolescents, including those related to health and eating. We report this case to highlight the influence of social media on pica-related behaviors and its potential for life-threatening complications.

We describe the case of an 18-year-old female with a past medical history of iron deficiency anemia and no formal psychiatric diagnosis, who developed a pattern of consuming tissue paper beginning around age 12. This behavior reportedly followed exposure to related content on social media and progressed to a compulsive pattern that led to colonic obstruction. At age 18, the patient required a right hemicolectomy due to an accumulation of undigested paper (xylobezoar). To our knowledge, this is the first reported case in which such a severe clinical outcome related to paper ingestion in an adolescent was directly associated with social media exposure.

This case highlights how behavioral patterns observed online may contribute to high-risk ingestion behaviors in vulnerable individuals. Clinicians may benefit from incorporating questions about social media use when evaluating patients with unexplained gastrointestinal symptoms or nutritional deficiencies. Increased awareness of these emerging behavioral triggers can inform early identification and intervention strategies.

## Introduction

Pica is a psychiatric condition characterized by the persistent consumption of non-nutritive substances for at least one month [1]. A variety of substances have been reported in patients with pica, including hair, dirt, ice, uncooked rice, starch, and dust, among others [2]. Xylophagia, a rare form of pica, involves the consumption of items derived from wood, such as tree branches or paper products [3]. A major complication of xylophagia is the formation of a xylobezoar, an accumulation of undigested paper in the gastrointestinal tract [4]. Xylobezoars can lead to severe, life-threatening gastrointestinal complications, including partial or complete intestinal obstruction and intestinal volvulus. These bezoars have been reported in the esophagus, stomach, sigmoid colon, and rectum. Initial symptoms of a xylobezoar often include abdominal pain, constipation, or hematochezia, particularly in patients with a history of anemia or pica [4-6]. While some cases have resolved with non-surgical management, one particularly complicated case involved a rectal xylobezoar that caused a large pneumoperitoneum, ischemic colitis, and cecal perforation. The patient required an emergent surgical laparotomy, followed by subtotal colectomy and ileostomy, but unfortunately died due to severe hemorrhage and subsequent cardiac arrest [4].

Patients with pica commonly have underlying nutritional deficiencies, such as iron deficiency anemia or hypozincemia [7], and it has been suggested that the compulsive cravings associated with pica may be linked to a perceived physiological need for these nutrients [1]. A review of the literature found that pica can present with a wide spectrum of neurological, gastrointestinal, renal, and pulmonary symptoms and complications [8]. In some cases, treating the underlying deficiency, such as through intravenous iron therapy, has led to resolution of pica symptoms [6]. However, there is limited discussion regarding the onset of pica symptoms and behaviors, particularly in relation to the influence of social media on adolescents.

A recent cross-sectional study by Yurtdaş-Depboylu et al. [9] investigated the relationship between social media addiction and disordered eating behaviors among adolescents in Turkey. The study included 1,232 high school students aged 14 to 18, and found that adolescents with high levels of social media addiction had significantly higher scores on measures of orthorexia nervosa, disordered eating attitudes, and body image dissatisfaction. Specifically, 43.5% of participants with high social media addiction were found to have orthorexic tendencies, compared to just 21.8% among those with low addiction scores. The findings suggest that excessive engagement with social media, particularly content related to health, fitness, and diet, may reinforce obsessive behaviors around food and body image. While the study did not examine pica or its subtypes directly, the demonstrated link between compulsive social media use and maladaptive eating behaviors raises important questions about its potential role in the emergence of rarer conditions such as pica and xylophagia, which remain critically under-researched [9].

We present the case of an 18-year-old female with xylophagia, an underlying iron deficiency anemia, and a history of consuming tissue paper after initially observing the behavior on YouTube and TikTok. This behavior eventually developed into a compulsive craving. To our knowledge, this case is the first to explicitly document the onset of xylophagia after exposure to social media content, highlighting an under-recognized pathway through which compulsive eating behaviors can be triggered in adolescents. This suggests that healthcare providers should consider social media influences when evaluating pica and related disorders, particularly given the widespread access and engagement adolescents have with these platforms, and the fact that these behaviors have potential for life-threatening complications, as seen with our patient. 

## Case presentation

An 18-year-old female with a history of iron deficiency anemia and no past psychiatric history presented with lower abdominal pain and nine episodes of non-bilious, non-bloody emesis. In the emergency department, she denied ingesting any foreign objects. She was hemodynamically stable and received intravenous morphine for pain management. Laboratory results showed an elevated white blood cell count, decreased hemoglobin, and increased lactic acid (Table 1). 

**Table 1:** Laboratory tests during this admission.

Laboratory test	Findings	Normal range
White blood cell count	12.2 x 10³/µL	3.5-10 x 10³/µL
Hemoglobin	10.1 g/dL	11.4-15.4 g/dL
Lactic acid	2.5 mmol/L	0.7-2 mmol/L

**Figure 1:**
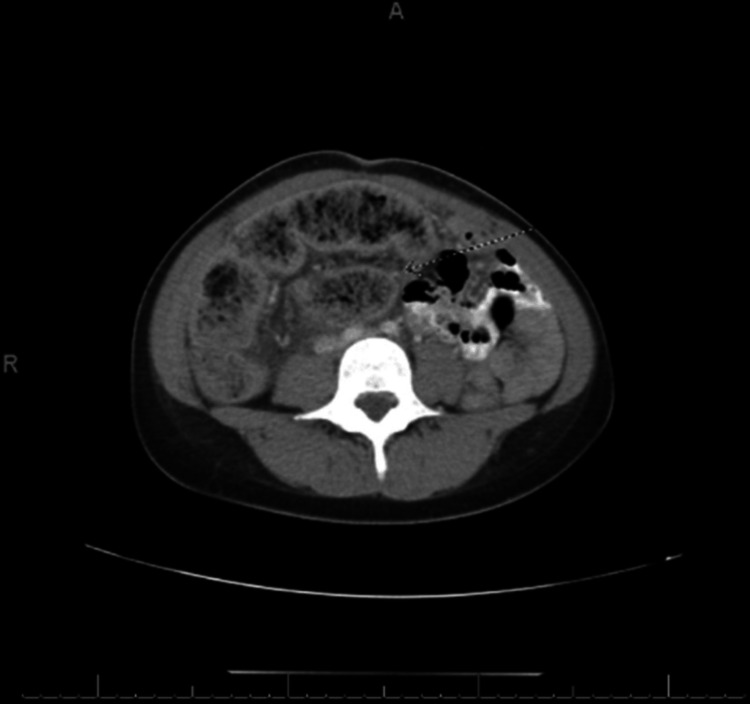
CT abdomen and pelvis demonstrating a cecal volvulus (arrow). CT, computed tomography

**Figure 2:**
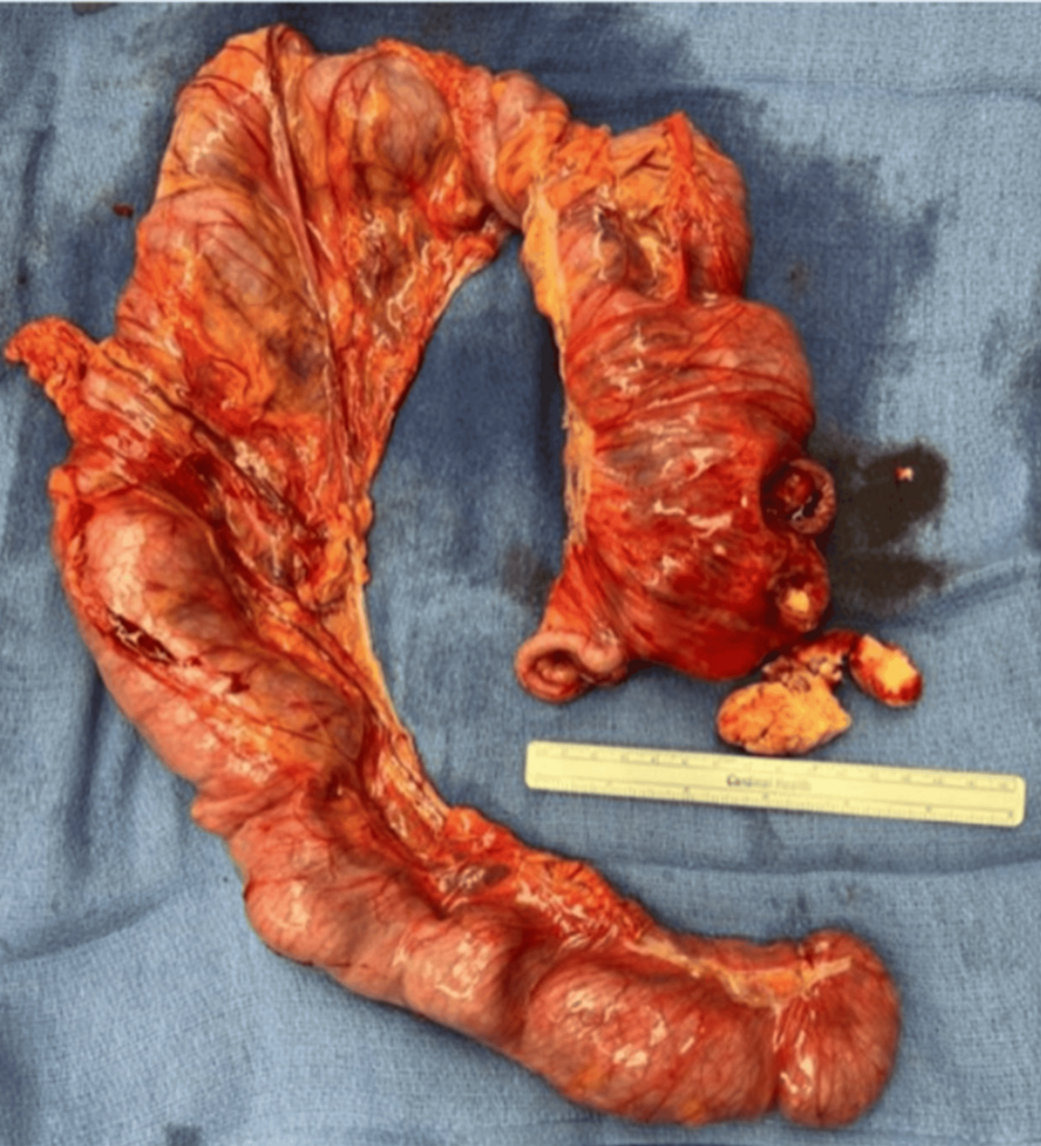
Surgical specimen from the right hemicolectomy. Paper-like material is shown adjacent to the specimen.

A computed tomography (CT) scan of the abdomen and pelvis revealed a mesenteric volvulus with an internal hernia obstructing the mid-transverse colon (Figure 1). The patient underwent exploratory laparotomy for colonic volvulus, and intraoperative findings included a dilated cecum and transverse colon with a cecal volvulus. An extended right hemicolectomy was performed. The resected colon specimen, opened intraoperatively, contained *paper-like material *(Figure 2). Psychiatry was consulted on postoperative day 2 due to concerns about an underlying psychiatric disorder.

During the initial psychiatric evaluation, the patient again denied ingesting any substances. However, she reported a previous hospitalization for severe abdominal pain following ingestion of *toilet paper*. She explained that at age 12, after watching someone on YouTube, she tried eating tissue paper and enjoyed the sensation. Over time, the behavior became a compulsive craving, leading her to consume up to one roll of toilet paper per sitting, often vomiting afterward, which temporarily relieved the craving. She even developed a preference for a specific brand of toilet paper. She denied anxiety, distress, or obsessions related to the behavior, and clarified she did not engage in this practice with others or as a means of weight control. No history of learning disabilities or substance abuse was elicited either. While she admitted to enjoying eating toilet paper in the past, she denied any recent consumption before her hospitalization. Although cravings persisted, she had stopped the behavior due to the abdominal pain it caused.

The patient had two prior emergency department visits for abdominal pain. At age 16, her mother discovered the tissue paper consumption after the patient admitted to trying it after seeing it on social media. Another visit occurred a year later at age 17, with abdominal pain and CT findings suggesting a partial small bowel obstruction. During the second visit, she was found to have an elevated white blood cell count and C-reactive protein (Table 2). At this point, the patient and her mother left against medical advice, and the patient disclosed participation in a *TikTok challenge* involving tissue paper consumption.

**Table 2:** Laboratory tests from previous emergency department visit.

Laboratory test	Findings	Normal range
White blood cell count	54.4 x 10³/µL	3.5-10 x 10³/µL
C-reactive protein	8.43 mg/dL	<1 mg/dL

Following the psychiatric evaluation, the patient was diagnosed with pica disorder. Recommendations included abstaining from consuming paper products and outpatient psychiatric follow-up. Although treatment for iron deficiency anemia was offered, the patient declined. She made a full recovery after her surgical resection, with no postoperative complications noted on her surgical follow-up 10 days after discharge. No further surgical follow-up was recommended. She has had no further clinical encounters in our healthcare system, including with psychiatry, as per review of the electronic medical record 18 months after she was discharged from the hospital.

## Discussion

Pica refers to the craving and consumption of non-food, inedible substances [3]. While this behavior is considered abnormal after the age of two [1], it's important to recognize that it may be culturally or religiously accepted in certain parts of the world, such as sub-Saharan Africa [10]. Pica tends to be more common in specific populations, including pregnant women, individuals from lower socioeconomic backgrounds, those with intellectual or developmental disabilities (such as autism), and people with psychiatric conditions like depression or schizophrenia [3,10].

There is a well-documented connection between pica and nutritional deficiencies, particularly iron deficiency anemia, though the exact nature of this relationship remains unclear. It is still debated whether the deficiencies drive the cravings or whether consuming non-nutritive items contributes to the deficiency. Clinically, pica should be distinguished from other eating disorders such as anorexia nervosa, bulimia nervosa, binge-eating disorder, and rumination disorder [1]. Obsessive-compulsive disorder can also present with repetitive ingestion behaviors. Some studies suggest involvement of the brain’s dopamine reward system, with iron deficiency linked to reduced dopamine receptor availability [10].

Importantly, symptoms of pica often improve when underlying nutritional deficiencies are corrected. For instance, Nemeth et al. reported a case of xylophagia in an adolescent that resolved following treatment of iron deficiency anemia [10]. However, many patients are hesitant to disclose these behaviors due to embarrassment, which can delay diagnosis. Regardless of the substance consumed, pica can result in serious medical complications, including esophageal injury, gastrointestinal obstruction, perforation, and, in some cases, death [11,12].

Given these risks, clinicians should maintain a high level of awareness and consider screening individuals at higher risk, including adolescents, people with intellectual disabilities, and patients with unexplained anemia. Routine, nonjudgmental questioning about unusual eating habits should be part of both psychiatric and medical assessments.

In one reported case, a patient who developed an esophageal obstruction after eating toilet paper explained that a friend had recommended it to help with diarrhea. This example illustrates how misinformation can influence behavior. With the rise of social media, such advice can now spread rapidly, increasing the potential for harmful imitation, particularly among adolescents [12].

In recent years, there has been a rise in *mass social media-induced illness*, where groups of adolescents present with symptoms they were exposed to online. Functional tics and behaviors mimicking dissociative identity disorder have become more common following trends on platforms like TikTok. These patterns have been described using terms such as *Munchausen’s by Internet* and *social media-associated abnormal illness behavior* [13].

Temporal associations between viral social media challenges and increases in the ingestion of substances like diphenhydramine and laundry pods further support the role of these platforms in influencing harmful behaviors [14]. Haltigan et al. describe social media as an incubator for new forms of behavioral psychopathology, many of which do not fit neatly into existing diagnostic categories. Pica may be one such behavior [15]. Preliminary studies also suggest that taking a break from social media can improve symptoms in individuals with eating disorders, hinting at potential benefits for other socially driven behaviors as well [16].

Considering these patterns, it is increasingly important for clinicians to ask about social media exposure when evaluating patients with unusual ingestion habits. Educational efforts directed at both patients and families may help counter misinformation and reduce risky behaviors. Preventive strategies might also include digital literacy education and closer collaboration between healthcare professionals, schools, and social media platforms. Further research is needed to better understand how online content influences health behaviors and to develop targeted interventions.

## Conclusions

These findings highlight the potential for social media to influence and propagate harmful behaviors among adolescents, including pica. Given these developments, it is essential for clinicians to prioritize obtaining a thorough history, especially in adolescents presenting with nonspecific symptoms such as abdominal pain, vomiting, constipation, or nutritional deficiencies. It is equally important to inquire about social media use and the potential influence of social media on their behaviors.
